# Redox signaling fidelity in immunosenescence and inflammaging: from oxidative stress to precision immune geroscience

**DOI:** 10.3389/fimmu.2026.1908166

**Published:** 2026-10-09

**Authors:** Wenjing Lu, Junqiang Yan

**Affiliations:** 1 Key Laboratory of Neuromolecular Biology, The First Affiliated Hospital, College of Clinical Medicine of Henan University of Science and Technology, Luoyang, China; 2 Department of Neurology, The First Affiliated Hospital, College of Clinical Medicine of Henan University of Science and Technology, Luoyang, China

**Keywords:** immunosenescence, inflammaging, innate immunity, mitochondrial dysfunction, mitophagy, precision immune geroscience, reactive oxygen species, redox signaling fidelity

## Abstract

Immunosenescence and inflammaging are accompanied by chronic inflammatory tone, impaired host-defense responses, defective resolution, and altered immunometabolic adaptation. Although these processes are often linked to oxidative stress, reactive oxygen species (ROS) also function as regulated immune signals. Here, we propose that aging immunity may involve not only increased oxidant burden but also a progressive loss of redox signaling fidelity, defined as the capacity of immune cells and tissues to generate, encode, transmit, decode, and resolve redox-dependent information with source specificity, spatially organized routing, temporal precision, biochemical reversibility, and proportionate downstream interpretation. We organize this framework into five information-flow layers: source-controlled reactive-species generation, reversible cysteine-centered and secondary-mediator encoding, spatially organized transmission, immune and inflammatory decoding, and resolution/reset by antioxidant, autophagy, mitophagy, lysosomal, and proteostatic systems. We then map age-associated fidelity loss onto T cells, B cells and hematopoietic progenitors, macrophages and microglia, neutrophils, dendritic cells, natural killer cells, regulatory T cells, and senescent-cell communication. The framework helps explain why related redox signals can support host defense, antigen-specific activation, and adaptive stress responses in one context, yet promote maladaptive NF-kappaB, NLRP3, cGAS-STING, interferon-linked inflammation, and senescence-associated secretory propagation when source coupling, timing, spatial routing, reversibility, decoder proportionality, or resolution fails. We also discuss why broad antioxidant suppression may underperform when it lowers oxidant abundance while flattening adaptive redox dynamics. Finally, we outline quantitative metrics, decisive experiments, controversies, and biomarker-guided therapeutic principles for precision immune geroscience.

## 1 Introduction: immune aging as redox information failure

The free radical theory of aging provided an important foundation for linking age-associated decline to oxidative injury in DNA, lipids, proteins, and membranes (1, 2). However, a damage-only model is too narrow for contemporary redox biology. ROS are not merely toxic metabolic by-products; they can act as regulated mediators of immune activation, metabolic remodeling, stress adaptation, and cell-fate decisions (3–7). This dual role is especially important in immunology, where ROS participate in receptor signaling, antimicrobial defense, antigen-driven activation, inflammasome regulation, inflammatory resolution, and broader physiological redox signaling (8, 9).

Immunosenescence and inflammaging make this duality clinically relevant. Older organisms often show reduced vaccine responsiveness, impaired pathogen clearance, altered T- and B-cell compartments, myeloid inflammatory bias, defective efferocytosis, and chronic low-grade inflammatory tone (10–14). Additional immune-aging studies link these phenotypes to T-cell remodeling, altered immune-metabolic inflammation, and impaired protective responses (15–20). Recent mechanistic and single-cell studies further connect aged immune systems to organ senescence, clonal GZMK+ T-cell states, and broader peripheral immune remodeling (21–24). Contemporary reviews also synthesize immunosenescence, inflammaging, immune resilience, and therapeutic targets (25–29). These phenotypes are frequently accompanied by oxidative stress, yet the key question is not only whether ROS increase with age. A more immune-relevant question is whether aged cells retain the ability to discriminate informative, stimulus-coupled redox signals from chronic redox noise.

We use the term redox signaling fidelity to describe this discriminative capacity as a relative, multidimensional property rather than a binary state. Higher-fidelity signaling does not imply perfectly noise-free, leak-free, or deterministic chemistry; physiological redox networks inevitably contain background activity, diffusion, stochastic variation, and pathway crosstalk. Instead, higher fidelity denotes comparatively better preservation of the relationship between a defined biological input and an appropriate redox-dependent output, whereas lower fidelity denotes reduced discriminability caused by source uncoupling, elevated or variable background activity, altered temporal patterning, spatial misrouting or delayed propagation, loss of reversible encoding, disproportionate decoding, or delayed resolution. Because superoxide, hydrogen peroxide, radical species, and enzyme-derived oxidants differ in lifetime, diffusivity, reaction selectivity, and biological targets, we use ‘ROS’ as an umbrella term only when the evidence does not support species-level assignment (6, 9, 30).

This Review has three aims. First, we define redox signaling fidelity as an operational and measurable property rather than a metaphor. Second, we reposition redox-fidelity loss as a framework for immunosenescence and inflammaging, with cell-type-specific consequences across adaptive and innate immunity. Third, we propose experimental and therapeutic principles in which interventions are evaluated by their ability to restore source specificity, reversible encoding, spatial routing, decoder coherence, and resolution kinetics rather than only by lowering bulk ROS abundance.

## 2 Scope and literature selection

This article is a critical narrative Review rather than a systematic review or meta-analysis. The literature was searched and updated through September 2026, focusing on primary mechanistic studies, influential reviews, and methodological papers relevant to redox signaling, immune aging, ROS measurement, cysteine redox proteomics, mitochondrial quality control, autophagy and mitophagy, innate immune decoding, inflammatory resolution, senescence, and redox-directed therapeutic strategies.

Search concepts included redox signaling, ROS dynamics, superoxide, hydrogen peroxide, oxidative stress, oxidative eustress, immunosenescence, inflammaging, mitochondrial ROS, NADPH oxidase, NAD+/NADH, NADP+/NADPH, cysteine oxidation, redox switches, redox proteomics, lipid peroxidation, 4-hydroxynonenal, itaconate, redox oscillations, circadian redox rhythms, NRF2, NF-kappaB, NLRP3, cGAS-STING, interferon signaling, mitophagy, autophagy, trained immunity, T-cell aging, B-cell immunosenescence, dendritic-cell aging, neutrophil oxidative burst, antioxidant therapy, PBMC processing, pre-analytical variability, assay reproducibility, and precision geroscience. We prioritized studies that addressed chemically or compartment-resolved reactive species, temporal redox dynamics, cysteine-centered redox encoding, secondary electrophilic or metabolic mediators, ROS-dependent immune activation, mitochondrial or NOX-derived signaling, quality-control pathways, limitations of nonspecific antioxidant therapy, and quantitative redox metrics.

Evidence is interpreted according to strength: direct aging evidence, indirect mechanistic evidence, correlative evidence, and unresolved gaps. This distinction is important because many aging studies measure oxidant abundance, whereas a fidelity framework requires dynamic, compartment-specific, input-output measurements. The goal is not to catalog all oxidative-stress findings in aging, but to distinguish redox burden from redox information quality in immune contexts.

## 3 Redox signaling fidelity: operational definition and immune relevance

Redox signaling fidelity is defined here as the degree to which redox dynamics preserve an interpretable relationship between an upstream physiological input and an appropriate downstream response. It is not synonymous with redox homeostasis, oxidative stress, oxidative eustress, or hormesis. Redox homeostasis emphasizes balance; oxidative stress emphasizes molecular injury; oxidative eustress and distress distinguish adaptive from damaging oxidant states; and hormesis emphasizes beneficial responses to low-level stress (8, 9, 31, 32). Fidelity adds an input-output dimension and is explicitly relative rather than absolute: no reactive-molecule signaling pathway is expected to be perfectly confined or noise-free. The relevant question is whether source identity, temporal structure, reversible chemistry, spatial routing, decoder behavior, and recovery remain sufficiently coupled to the initiating biological input to preserve signal discriminability.

The concept also differs from adjacent frameworks such as the redox code (33). The redox code provides a broad biochemical framework for understanding the organization of redox networks, whereas redox signaling fidelity focuses on whether that organization remains accurate during aging and immune activation. A redox event may be detectable but poorly informative if it is generated from the wrong source, diffuses outside the relevant microdomain, becomes chemically irreversible, or activates a decoder that no longer reflects the initiating stimulus.

In operational terms, a higher-fidelity immune redox response depends on five linked features. Source specificity means that reactive-species production is coupled to a defined generator, such as a mitochondrial respiratory site, a NOX complex, an oxidoreductase system, or a peroxisomal reaction (34–41). Encoding means that reactive chemistry is converted into reversible or otherwise interpretable molecular changes, including cysteine oxidation, S-glutathionylation, disulfide formation, relay reactions, and selected secondary electrophilic/metabolic signals (30, 33, 42–45). Transmission requires sufficiently selective routing and relay kinetics to favor biologically appropriate target engagement without assuming impermeable compartment boundaries. Decoding requires redox-sensitive pathways to translate the signal into transcriptional, metabolic, immune, or inflammatory outputs. Resolution resets the system through buffering, repair, autophagy, mitophagy, lysosomal clearance, and proteostasis (46–53).

Fidelity can deteriorate even when total ROS levels are not markedly elevated. Conversely, ROS flux can be substantial but physiologically informative if its source, timing, reversibility, and decoding remain intact. A fidelity framework therefore asks whether an aged immune cell can still interpret ROS, not only whether it accumulates more oxidized molecules (**Box 1**).

Box 1 **Operational criteria for demonstrating redox-fidelity loss in immune aging.**

A strong demonstration of redox-fidelity loss should show linked defects within the same immune model rather than an increase in bulk ROS alone. Useful evidence would include: (1) altered stimulus-linked dynamics despite similar mean oxidant abundance, such as reduced pulse amplitude, elevated baseline variance, abnormal phase or rhythmicity, or delayed recovery; (2) reduced coupling between a defined physiological input and a chemically or source-resolved redox output; (3) impaired reversibility, such as persistence of normally reversible cysteine states; (4) abnormal spatial routing, including excessive off-target spread or insufficient/delayed propagation to an intended target; (5) decoder mismatch, such as blunted NRF2 or AMPK responses or disproportionate NF-kappaB, NLRP3, cGAS-STING, or interferon-linked activation; and (6) delayed resolution measured by return-to-baseline kinetics, mitophagy/autophagy flux, cytokine resolution, or restoration of signal-to-background discrimination. The strongest evidence would show that a targeted intervention restores one or more of these input-output relationships and immune function without requiring global suppression of ROS. Conversely, increased oxidative damage with preserved stimulus-specific dynamics and decoding should be classified primarily as oxidative stress rather than redox-fidelity loss.

### 3.1 Quantitative readouts of redox signaling fidelity

To make the concept experimentally useful, redox signaling fidelity should be reported through paired dynamic and functional readouts rather than by bulk oxidant abundance alone. A source-coupling metric can describe the relationship between a prespecified input and a redox output attributed, with orthogonal evidence, to a specified generator such as a respiratory-chain site or NOX complex. It is not intended as a universal scalar: source perturbation can simultaneously alter ATP production, metabolites, calcium handling, membrane potential, or other immune-relevant functions, particularly in mitochondria. Interpretation therefore requires orthogonal source perturbation, matched metabolic and viability controls, and downstream functional readouts. Redox signal-to-noise ratio (SNR) should likewise be defined prospectively, for example as a stimulus-induced peak or integrated response relative to pre-stimulus variability rather than simply as ‘high’ or ‘low’ ROS.

Here, redox noise denotes background redox activity that is insufficiently or unreliably coupled to the physiological input of interest and therefore reduces signal discriminability. Its experimental denominator must be specified and may include within-cell temporal baseline variance, cell-to-cell heterogeneity, off-target compartment signals, source-uncoupled activity, or a prespecified combination of these components. A reversibility index can quantify the fraction and half-time of a reversible cysteine or relay state returning toward baseline after stimulation. A spatial-routing/propagation index can compare intended-target versus off-target signals and, where feasible, propagation delay. Decoder proportionality can relate defined redox inputs to NRF2, AMPK/mTOR, NF-kappaB/NLRP3, cGAS-STING/interferon, or cytokine outputs across dose and time, while recognizing that correlation alone does not establish redox causality. Finally, a resolution half-time can quantify return of redox, inflammatory, and functional outputs toward baseline.

These measurements generate falsifiable predictions. Aged immune cells may show reduced stimulus-linked SNR, altered pulse or rhythmic structure, slower reversibility, excessive spatial spread or delayed propagation, and less coherent decoder responses than young cells under matched inputs. Conversely, an intervention that restores fidelity should improve one or more prespecified dimensions of coupling, reversibility, temporal organization, routing, decoder coherence, or resolution even if mean ROS abundance changes only modestly. Importantly, not every dimension is expected to fail in every aged cell type; the framework is intended to identify the dominant defect in a defined biological context.

### 3.2 Temporal redox fidelity: pulses, oscillations, circadian rhythms, and recovery

Temporal fidelity should not be reduced to a single concept of ‘ROS oscillation.’ At least four temporal regimes can be distinguished experimentally: stimulus-evoked pulses on seconds-to-minutes timescales, recurrent oscillatory behavior, circadian or diurnal redox rhythms, and post-stimulus recovery kinetics. Loss of temporal fidelity can therefore appear as reduced or excessive amplitude, an elevated baseline, increased aperiodic variability, altered frequency or phase, damping or loss of rhythmicity, prolonged signal duration, or delayed return to baseline. Similar time-averaged ROS levels can consequently conceal very different signaling states. Mitochondrial ROS-linked oscillations have been observed experimentally in excitable cells, while peroxiredoxin oxidation cycles provide evidence for conserved approximately 24-hour redox rhythms, including in anucleate human erythrocytes (54–56).

More direct evidence links endogenous H_2_O_2_ rhythms to mammalian clock function: diurnal H_2_O_2_ oscillations can reversibly modify CLOCK and influence circadian timing, whereas metabolic/redox perturbations can preferentially alter clock phase or amplitude rather than period (57, 58). Importantly for aging, a 2026 study reported widespread age-associated disruption of redox oscillations across mouse tissues and showed that timed restoration of redox rhythms improved several aging-related phenotypes; perturbation of redox-sensitive CLOCK Cys195 produced aging-relevant reprogramming (59). These data support temporal organization as a plausible fidelity dimension but do not establish a universal mechanism in immune cells. The direction of causality is likely bidirectional: circadian deterioration can alter metabolism and redox state, while redox perturbation can feed back onto clock machinery. Direct young-versus-aged, immune-cell-resolved studies remain a priority.

## 4 Architecture of immune redox information flow

For analytical clarity, redox signaling can be organized into generation, encoding, transmission, decoding, and resolution/reset (**Figure 1**). Generation describes production by defined sources. Mitochondrial ROS arise from respiratory-chain sites and depend on substrate use, membrane potential, electron flow, and local antioxidant capacity (34, 35, 37, 38). NOX-derived oxidants participate in receptor-dependent immune, vascular, and stress signaling (36, 60, 61). Peroxisomes, ER oxidoreductases, monoamine oxidases, myeloperoxidase-containing systems, and related enzymes can also shape localized redox chemistry. The umbrella term ROS is chemically heterogeneous: superoxide, hydrogen peroxide, radical species, and enzyme-derived oxidants differ substantially in lifetime, membrane permeability, diffusivity, target selectivity, and reaction kinetics (6, 9, 30). Thus, source fidelity includes both the generator and, where experimentally resolvable, the identity of the reactive species produced.

**Figure 1:**
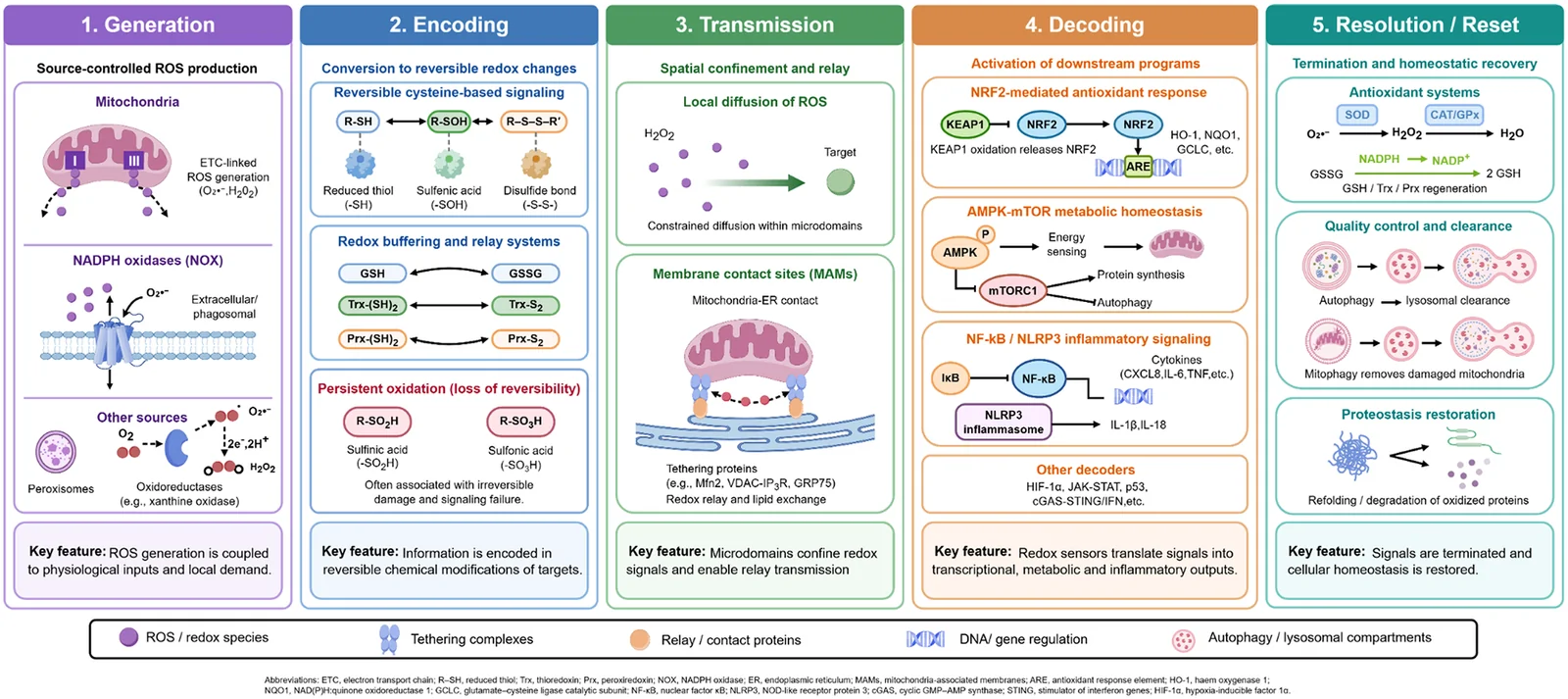
Immune-relevant architecture of higher-fidelity redox information flow. Redox signaling fidelity can be organized into generation, encoding, transmission, decoding, and resolution/reset, spanning source- and species-aware reactive chemistry, reversible cysteine-centered and secondary-mediator encoding, spatially organized transmission, immune and inflammatory decoding, and antioxidant/autophagy/mitophagy/proteostasis-mediated resolution. Higher fidelity is relative and does not imply perfectly noise-free or spatially confined signaling. This is an original conceptual schematic; no experimental data are shown. Created using Figdraw.

Metabolic redox couples provide an upstream and parallel layer of this architecture. NAD+/NADH is closely linked to substrate oxidation, glycolysis, and mitochondrial electron transfer, whereas NADP+/NADPH supplies reducing equivalents for glutathione-, thioredoxin-, and peroxiredoxin-dependent antioxidant/relay systems and also supports NOX activity. These pools are compartmentalized and dynamically coupled to immunometabolism (62). They should not be treated as interchangeable with ROS; rather, altered NAD(H) or NADP(H) state can change both reactive-species generation and the capacity to buffer, relay, and terminate redox signals. In aging, loss of metabolic flexibility may therefore reduce redox signal discriminability even when mean ROS abundance changes only modestly.

At the encoding stage, ROS are converted into reversible biochemical information. Cysteine residues are especially important because their reactivity depends on pKa, solvent accessibility, local electrostatics, and proximity to ROS-generating sites (30, 42, 63, 64). Peroxiredoxin, thioredoxin, and glutathione systems can function as redox relays, allowing hydrogen peroxide signals to be transferred selectively rather than to diffuse indiscriminately. Encoding is lost when reversible modifications are replaced by persistent lesions, including irreversible protein oxidation, lipid peroxidation adducts, or glycoxidative modifications.

Redox information is also carried by secondary electrophilic and metabolic mediators rather than by protein oxidation alone. Lipid peroxidation-derived aldehydes such as 4-hydroxynonenal (4-HNE) can covalently modify signaling proteins, including KEAP1-associated sensor pathways (45, 65), and can modulate inflammasome output in macrophage systems (44). Their persistence, intracellular movement, and effective signaling range are context dependent and should not be inferred from a simple comparison with primary reactive oxygen species. Itaconate should be distinguished mechanistically from a direct ROS oxidation product: it is an immunometabolite generated in activated macrophages that can alkylate KEAP1 cysteines and activate NRF2-linked anti-inflammatory programs (43, 65). Such secondary mediators can add downstream signaling layers that do not necessarily track with bulk ROS measurements or nonspecific antioxidant exposure.

Transmission entails movement of redox information across subcellular and intercellular distances. Local hydrogen peroxide diffusion, membrane permeability, organelle contact sites, redox relay proteins, secondary electrophiles/metabolites, cytokines, and extracellular vesicles can all participate (43–45, 66, 67). In young or resilient cells, the balance among local generation, consumption, relay chemistry, membrane properties, and contact-site architecture biases reactions toward appropriate targets without creating perfectly sealed boundaries. In aging, transmission may fail in opposite directions: excessive spread or misrouting can reduce spatial selectivity, whereas insufficient or delayed propagation can prevent timely target engagement. Section 5.3 develops this bidirectional model and its measurement limitations.

Decoding converts redox inputs into immune decisions. NRF2, AMPK, mTOR, NF-kappaB, HIF-1alpha, NLRP3, cGAS-STING, and interferon-linked programs can all act as redox-sensitive decoders, although their relative importance varies by cell type, stimulus, disease context, and timing (14, 68, 69). cGAS-STING and interferon-linked outputs are especially relevant when redox failure intersects with DNA danger signaling (53, 70, 71). After decoding, resolution is the final functional layer. Buffering systems reset reversible oxidation, proteostasis systems repair or remove damaged proteins, and quality-control pathways reduce persistent ROS-generating substrates (46–50). Autophagy and mitophagy help eliminate persistent ROS-generating sources and prevent previous signals from becoming background noise (51–53).

## 5 How aging may degrade immune redox signaling fidelity

The proposed transition from comparatively organized, input-coupled redox signaling to lower-fidelity signaling during aging is summarized in **Figure 2**. Although individual tissues and immune compartments differ, five recurrent defect classes are especially relevant to immunosenescence and inflammaging.

**Figure 2:**
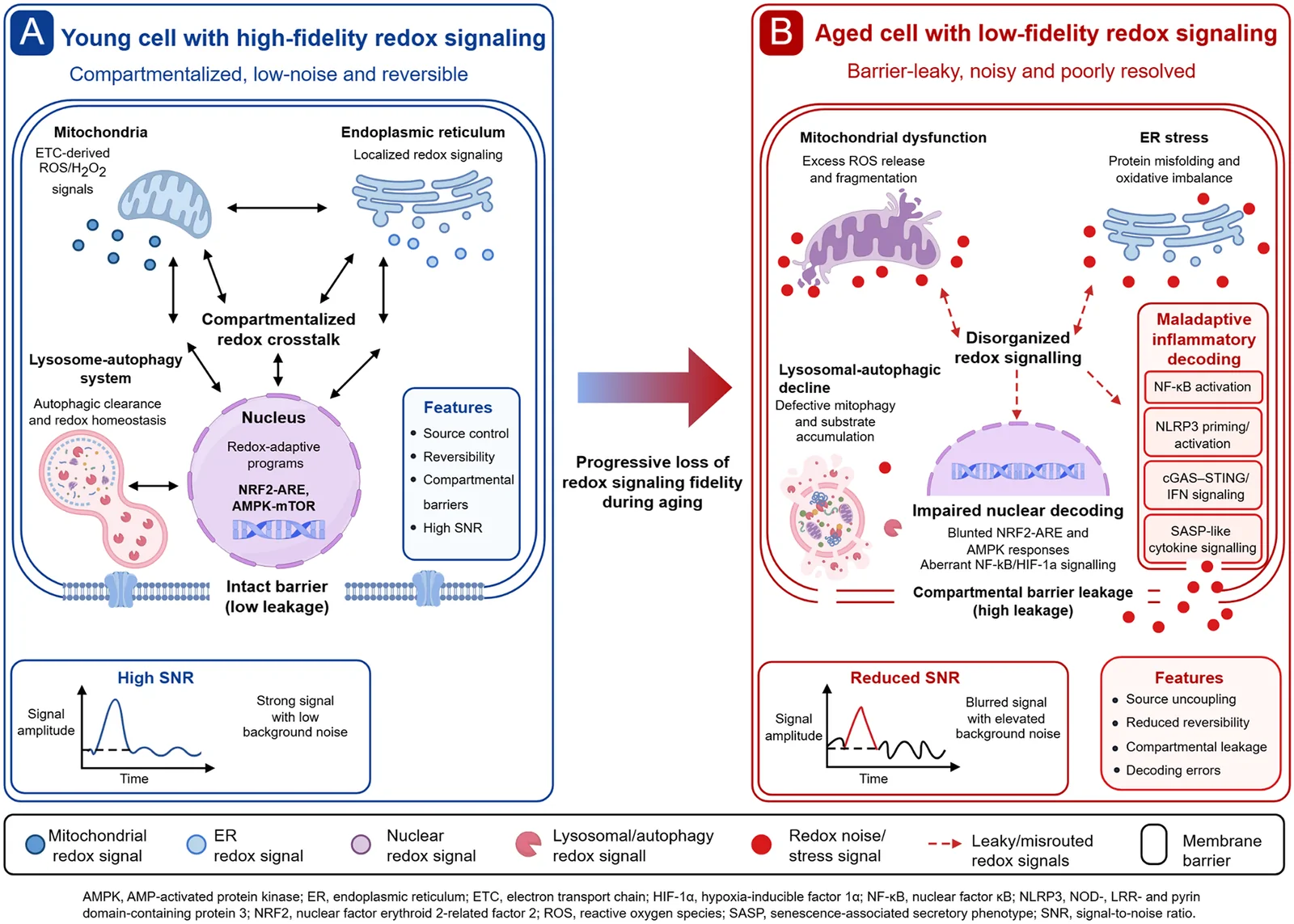
Redox-fidelity loss in immunosenescence and inflammaging. **(A)** Young cells preserve comparatively organized, high signal-to-background redox communication. **(B)** Aging may promote mitochondrial dysfunction, ER stress, defective mitophagy, lysosomal decline, altered barrier/contact-site behavior, and maladaptive inflammatory decoding. The schematic illustrates a leakage/misrouting-dominant failure mode; spatial transmission can also fail through insufficient or delayed propagation, which is not separately depicted. The image summarizes representative aging-associated changes and does not imply that all features are universally present in every aged cell type. This is an original conceptual schematic; no experimental data are shown. Created using Figdraw.

### 5.1 Source distortion and loss of stimulus coupling

Aging alters the behavior of redox-generating sources. Mitochondrial dysfunction changes substrate use, electron-flow efficiency, membrane potential, and organelle dynamics, increasing the likelihood that mitochondrial ROS behave as stochastic leakage rather than structured metabolic information (72–76). Mitochondrial ROS production also depends on respiratory-chain site, electron flow, and hydrogen peroxide handling (37–40). Recent inflammation-and-aging reviews extend these mitochondrial defects to immune dysregulation (77, 78). NOX systems may also become dysregulated in inflammatory and vascular contexts, introducing receptor-independent or poorly resolved ROS production (36, 60, 61). In immune cells, source distortion reduces the informational value of ROS because the signal no longer reliably reports antigenic stimulation, pathogen sensing, phagosome maturation, or metabolic state.

Evidence status: source distortion is supported by strong mitochondrial and NOX pathway evidence, but direct young-versus-aged input-output studies that quantify source coupling, SNR, and downstream immune decoding in the same cells remain limited.

### 5.2 Encoding failure and loss of reversibility

Physiological redox signaling relies on reversible chemistry. During aging, antioxidant buffering capacity may decline, redox relays may become oxidatively compromised, and reactive cysteine networks may become less responsive to new inputs. The practical result is a shift from reversible modifications toward persistent lesions such as protein carbonylation, lipid peroxidation adducts, and glycoxidative products (42, 79–81). Redox proteomic studies provide tools to distinguish reversible cysteine proteoforms from persistent damage (63, 64). Persistent lesions are poor information carriers because they cannot be rapidly reversed. This distinction separates redox signaling from oxidative damage.

Evidence status: redox proteomics has strengthened residue-level mapping of cysteine-centered modifications and can distinguish selected reversible redox states from persistent lesions, but conventional bulk workflows provide limited information about the precise subcellular origin or rapid kinetics of the initiating event. Proximity-labeling and organelle-enrichment strategies can improve localization, yet remain imperfect and do not by themselves establish causal signaling (63, 64, 82). Thus, proteomic changes should be interpreted together with dynamic sensors, source perturbation, and functional experiments.

### 5.3 Transmission failure: misrouting, excessive spread, or impaired propagation

High-fidelity transmission means appropriate delivery of redox information, not maximal physical confinement. Effective H_2_O_2_ and related redox gradients emerge from the balance among local production, consumption, membrane permeability, scavenging, relay chemistry, and target reactivity; a signal can therefore fail through excessive spread/misrouting or through insufficient or delayed propagation. Aging alters membrane composition, lipid oxidation, organelle morphology, vesicular traffic, and contact-site architecture (79, 80, 83). It may also alter the physical state of the cytoplasm: recent measurements in aged mouse-derived cells identified increased cytoplasmic crowding and stiffness with reduced intracellular movement (84). These observations support a bidirectional model in which aging can either blur spatial selectivity or impede timely target engagement. Consequently, microdomain localization should be treated as a context-dependent experimental hypothesis rather than an assumed property of all ROS signals.

Evidence status: redox microdomain imaging and contact-site biology support spatially structured signaling, but current methods rarely reconstruct the complete trajectory of a reactive species with simultaneous chemical, spatial, and temporal resolution. Direct aging studies that distinguish excessive spread from slowed or failed propagation remain sparse. Spatial-fidelity claims should therefore specify the measured species or redox reporter, its target compartment, relevant kinetics, and the alternative transport/consumption explanations that were tested.

### 5.4 Decoder inflexibility and inflammatory bias

Aged cells may fail to decode redox inputs proportionally. NRF2 responses can become attenuated or delayed, AMPK/mTOR balance may shift in ways that reduce metabolic flexibility, NF-kappaB may remain chronically active, and inflammasomes may become primed (14, 68, 69, 71, 85). Thus, the same ROS pulse that supports adaptation in a young immune cell may be ignored, exaggerated, or misclassified in an aged cell. Decoder inflexibility provides a mechanistic bridge between oxidative stress, immune dysfunction, and inflammaging.

Evidence status: pathway-specific data are strong, but the proportionality between a defined redox input and a defined decoder output remains insufficiently measured in aging immune models.

### 5.5 Resolution failure

Resolution is the reset step that keeps a redox signal transient. Autophagy and mitophagy are therefore not merely degradative pathways; they are redox-resolution systems. By removing damaged mitochondria, oxidized cargo, and dysfunctional macromolecular assemblies, they reduce persistent background ROS and restore dynamic range (46–50). Autophagy and mitophagy also restrain persistent inflammatory signaling (51–53). Age-associated impairment of lysosomal function, cargo recognition, and mitophagic flux can convert transient redox perturbations into unresolved immune stress.

Evidence status: mitophagy and autophagy decline are well-supported features of aging, and recent work has directly linked mitophagy to suppression of cytosolic mtDNA-dependent cGAS-STING inflammation during aging (53). More integrated studies should test whether restoring mitophagy improves redox SNR and immune function simultaneously.

## 6 Immune-cell-specific manifestations of redox-fidelity loss

Immune aging offers a stringent test of the redox-fidelity framework because immune cells require ROS for receptor signaling, antimicrobial defense, metabolic reprogramming, differentiation, inflammatory resolution, and tissue repair. They are also vulnerable when redox signals become chronic, mislocalized, or poorly decoded (10, 14, 86–88). Adaptive and innate immune aging also involve T-cell remodeling, altered vaccine responsiveness, and immune-metabolic inflammation (15–19). Additional cell-type-specific literature supports these examples across T cells, B cells, dendritic cells, NK cells, Tregs, NLRP3-linked inflammation, trained immunity, and single-cell immune aging (20, 89–95). Recent single-cell and systems studies extend this view to clonal cytotoxic T-cell states and age-related blood immune remodeling (22–24). **Table 1** summarizes how different immune compartments may express distinct fidelity defects.

**Table 1:** Immune-cell-specific manifestations of age-associated redox-fidelity loss and testable readouts.

Immune compartment	Physiological redox signal	Aging-associated fidelity defect	Dominant decoder/output	Testable readout
T cells	TCR-linked mitochondrial ROS supporting activation, calcium signaling, and metabolic adaptation.	Higher basal redox noise, weaker stimulus-coupled bursts, mitochondrial inflexibility, and reduced antigen discrimination.	NFAT/NF-kappaB, NRF2, AMPK-mTOR, cytokine and exhaustion programs.	TCR-induced compartment ROS SNR, mitochondrial function, cytokines, proliferation, exhaustion markers, vaccine-like activation.
B cells and hematopoietic progenitors	BCR-linked redox remodeling, mitochondrial adaptation, and low ROS states supporting stem/progenitor quiescence.	Impaired class switching/affinity maturation, inflammatory B-cell skewing, lineage bias, clonal selection, and exhaustion.	BCR signaling, NRF2/AMPK-mTOR, plasma-cell differentiation and antibody programs.	BCR-induced ROS, germinal-center response, antibody quality, class-switch recombination, HSPC ROS and lineage output.
Macrophages and microglia	TLR- and phagocytosis-linked mitochondrial/NOX ROS supporting bactericidal activity and inflammatory integration.	Chronic priming, defective efferocytosis, mitochondrial leakage, and impaired resolution.	NF-kappaB, NLRP3, cGAS-STING/interferon, cytokines and tissue-repair programs.	Stimulus-coupled mitochondrial and phagosomal ROS, IL-1beta/TNF/IFN output, efferocytosis, mitophagy and return-to-baseline kinetics.
Neutrophils	NOX2-dependent phagosomal oxidative burst coordinated with chemotaxis, degranulation, killing, and NETosis.	Mislocalized extracellular ROS, altered burst timing, impaired pathogen targeting, and tissue-damaging leakage.	Antimicrobial killing, NETosis, chemotaxis, and inflammatory tissue injury.	Real-time phagosomal versus extracellular ROS, burst timing, NET formation, chemotaxis, pathogen clearance, tissue injury.
Dendritic cells	TLR-linked redox signals supporting antigen uptake, maturation, interferon production, and T-cell priming.	Impaired antigen presentation, altered TLR response, low antiviral SNR, and cytokine imbalance.	IRF/interferon, NF-kappaB, antigen-presentation and tolerance programs.	TLR-induced ROS dynamics, type I IFN, IL-12/IL-10 balance, antigen presentation, T-cell priming.
NK cells and Tregs	Target-recognition, cytotoxic, suppressive, and tissue-repair functions coupled to metabolic-redox state.	Altered cytotoxicity or suppression, mitochondrial stress, senescent-cell surveillance defects, and context-dependent immune restraint.	Cytotoxic granule release, IFN-gamma/TNF, FOXP3-linked suppressive and tissue-repair programs.	Target-induced ROS/metabolic flux, cytotoxicity, suppressive capacity, senescent-cell clearance, resolution kinetics.

BCR, B-cell receptor; IFN, interferon; NK, natural killer; NOX, NADPH oxidase; NRF2, nuclear factor erythroid 2-related factor 2; ROS, reactive oxygen species; SNR, signal-to-noise ratio; TCR, T-cell receptor; TLR, Toll-like receptor; Treg, regulatory T cell.

### 6.1 T cells: loss of stimulus-coupled redox discrimination

T cells provide a clear example of stimulus-coupled redox signaling. Antigen-specific activation depends on coordination among T-cell receptor engagement, calcium signaling, mitochondrial metabolism, and ROS-sensitive transcriptional programs. Mitochondrial ROS support antigen-specific T-cell activation and metabolic adaptation (86). With age, naive T-cell pools contract, memory and exhausted phenotypes expand, mitochondrial flexibility declines, and inflammatory background noise increases (10, 15–18). Human T-cell aging and vaccine-related literature further emphasizes reduced diversity, exhaustion, clonal GZMK+ states, and impaired protective responses (19, 20, 22, 23). From a fidelity perspective, aged T cells may show both weaker stimulus-coupled redox bursts and higher basal noise, reducing their ability to distinguish antigen-driven signals from chronic inflammatory stress.

### 6.2 B cells and hematopoietic progenitors: redox control of immune renewal

B-cell activation, antibody production, germinal-center responses, and plasma-cell differentiation require metabolic and redox remodeling. B-cell immunosenescence is associated with reduced antibody quality, altered class-switch recombination, expansion of inflammatory or age-associated B-cell subsets, and weaker vaccine responses (89). Recent experimental work further suggests that B cells can directly drive CD4 T-cell immunosenescence and age-associated health decline (96). From a fidelity perspective, the critical issue is whether B-cell receptor-linked ROS dynamics remain coupled to antigenic stimulation, reversible thiol encoding, mitochondrial adaptation, and transcriptional programs that support affinity maturation. Aging may weaken this coupling by increasing inflammatory background noise, reducing mitochondrial flexibility, and altering the redox state of signaling and metabolic enzymes.

Hematopoietic stem and progenitor cells are also redox-sensitive and are influenced by inflammatory exposure during aging and clonal selection (97, 98). Low and well-buffered ROS states help maintain quiescence, whereas persistent or poorly resolved ROS can promote lineage bias, clonal selection, and exhaustion. Thus, redox-fidelity loss should be evaluated upstream of mature immune-cell dysfunction by testing whether ROS dynamics still guide quiescence, self-renewal, and lineage choice rather than simply by measuring whether ROS abundance is high or low.

### 6.3 Macrophages and microglia: biased inflammatory decoding

Macrophages and microglia illustrate how host-defense ROS can become sterile inflammatory noise. Macrophages use ROS for antimicrobial responses and inflammatory signal integration. TLR signaling can recruit mitochondrial ROS to enhance bactericidal activity (87), whereas mitochondrial dysfunction can promote inflammasome activation and cytokine production (88). Aging pushes macrophages and microglia toward chronic inflammatory tone, impaired efferocytosis, defective metabolic plasticity, and altered tissue repair. Fidelity loss in these cells is best conceptualized as biased decoding: NF-kappaB, NLRP3, and interferon-linked programs become more readily activated and less readily resolved (12–14, 69, 71). NLRP3-linked functional decline and trained-immunity programs provide additional mechanisms for chronic inflammatory memory (93, 94). Recent work suggests that aged bone-marrow macrophage extracellular vesicles can propagate paracrine senescence systemically (99).

### 6.4 Neutrophils: burst timing versus bulk oxidant output

Neutrophils show why oxidant quantity alone is an insufficient metric (100, 101). Their antimicrobial function depends on a tightly timed and spatially restricted NOX2-dependent oxidative burst within phagosomes, coordinated with chemotaxis, degranulation, microbial killing, and, in some contexts, NET formation. A large burst does not guarantee high fidelity if timing, phagosomal localization, granule coordination, or microbial targeting is impaired. Conversely, reduced pathogen-directed burst capacity may coexist with extracellular or tissue-damaging basal leakage. Direct testing therefore requires real-time burst kinetics, phagosomal versus extracellular ROS measurement, localization-resolved probes, and linked functional readouts of chemotaxis, pathogen clearance, NETosis, and tissue injury.

### 6.5 Dendritic cells, natural killer cells, and regulatory T cells: antigen presentation, surveillance, and tolerance

Dendritic cells integrate pathogen sensing, antigen presentation, cytokine production, and tolerance. Aging can impair dendritic-cell antigen uptake, migration, TLR responsiveness, interferon production, and cytokine balance (90). A redox-fidelity interpretation predicts that aged dendritic cells may misclassify redox cues by generating insufficient stimulus-coupled signals for antiviral or vaccine responses while sustaining inflammatory basal output.

Natural killer cells and regulatory T cells provide complementary examples of immune surveillance and immune restraint. NK-cell aging involves altered phenotype, cytotoxicity, cytokine production, and interactions with senescent-cell burden (91). Regulatory T cells may increase or change function with age, contributing to altered tolerance, tissue repair, or immune suppression depending on context (92). In both lineages, redox-fidelity loss should be tested by measuring whether ROS dynamics remain linked to target recognition, metabolic activation, cytotoxic or suppressive function, and timely resolution rather than by measuring total ROS alone.

### 6.6 Senescence, SASP, extracellular vesicles, and tissue-level redox noise

Senescent cells can convert intracellular redox infidelity into tissue-level noise. Mitochondrial dysfunction, DNA damage signaling, and the senescence-associated secretory phenotype can amplify redox and inflammatory signals beyond the initiating cell (102–105). Cytokines, extracellular vesicles, oxidized metabolites, and damage-associated molecular patterns can propagate low-fidelity redox states to neighboring cells, generating feed-forward links between local redox dysfunction and systemic inflammaging (11, 12, 66, 67, 106). Systems-level inflammaging and immune resilience literature further supports this cell-to-tissue propagation model (13, 14, 29).

## 7 Innate immune decoders and inflammatory amplification

The clearest immune decoders of redox infidelity are NF-kappaB, NLRP3, cGAS-STING, and interferon-linked pathways. NF-kappaB integrates cytokine, TLR, metabolic, and stress inputs. NLRP3 responds to mitochondrial dysfunction, ion fluxes, lysosomal damage, and redox-related danger signals, and canonical NLRP3 signaling has been linked to systemic low-grade inflammation and functional decline in aging (88, 93, 107). cGAS-STING connects mitochondrial or nuclear-derived DNA danger signals to interferon programs, and aging-focused work links impaired mitophagy and mtDNA release to cGAS-STING inflammation (53, 69, 70, 108). Persistent interferon-linked programs have also been conceptualized as IFN-aging (109).

These pathways are not pathological by default. They are essential for antimicrobial defense, antiviral responses, tissue repair, and immune surveillance. The fidelity problem arises when activation becomes disproportionate, chronically primed, spatially misrouted, or poorly resolved. Aged cells may therefore show inflammatory decoding errors: insufficient adaptive NRF2 or AMPK responses, excessive NF-kappaB or NLRP3 activation, and persistent interferon-linked signaling in the absence of an appropriate acute threat.

This framework also connects with trained immunity. Innate immune memory requires durable metabolic and epigenetic reprogramming that can enhance host defense but may also contribute to chronic inflammatory tone when poorly regulated (94). From a redox-fidelity perspective, trained immunity is potentially adaptive when redox inputs are well coupled to metabolic and epigenetic programs, but maladaptive when persistent redox noise sustains inflammatory memory without a resolvable stimulus.

## 8 Redox fidelity and the hallmarks of aging

Redox signaling fidelity can serve as a cross-cutting interpretive lens for several hallmarks of aging. Mitochondrial dysfunction represents source distortion. Loss of proteostasis, autophagy decline, and lysosomal dysfunction represent impaired resolution. Deregulated nutrient sensing reflects decoder imbalance among AMPK, mTOR, insulin/IGF, and stress-response pathways. Cellular senescence and inflammaging represent redox-noise propagation across cells and tissues (14, 105, 110, 111). Recent broad reviews place these processes within inflammation-aging and immunosenescence pathways (25, 78). Epigenetic drift may reflect, in part, corrupted coupling between redox inputs and transcriptional memory.

This interpretation should not be overextended. Genomic instability, telomere attrition, stem-cell exhaustion, altered intercellular communication, dysbiosis, and chronic inflammation intersect with redox biology but are not reducible to ROS biology. The value of the fidelity framework lies in its integrative utility: it helps explain why age-associated phenotypes often cluster together while preserving space for non-redox mechanisms.

Evidence status: redox biology intersects with multiple hallmarks of aging, but the fidelity model should be considered a testable organizing framework rather than a complete explanation for aging. Its strongest application is likely to be in contexts where mitochondrial quality control, immune activation, inflammatory propagation, and adaptive resilience are central.

## 9 Why poorly targeted antioxidant strategies may fail to restore immune redox fidelity

The inconsistent efficacy of antioxidant interventions is one of the strongest arguments for distinguishing redox burden from redox fidelity. Broad, poorly timed, or excessive ROS scavenging can lower oxidative-stress markers while also attenuating transient redox signals required for adaptive stress responses (31, 32, 112–114). However, physiological temporal organization is not restricted to a universal sinusoidal ROS pattern. Depending on context, informative redox dynamics may appear as isolated pulses, recurrent oscillations, circadian rhythms, or appropriately timed recovery. Experimental studies of endogenous H_2_O_2_ and peroxiredoxin rhythms, together with recent evidence that aging disrupts redox rhythmicity, support this broader temporal principle (55–59). **Figure 3** therefore illustrates one possible oscillatory pattern rather than a claim that all immune redox signals are oscillatory.

**Figure 3:**
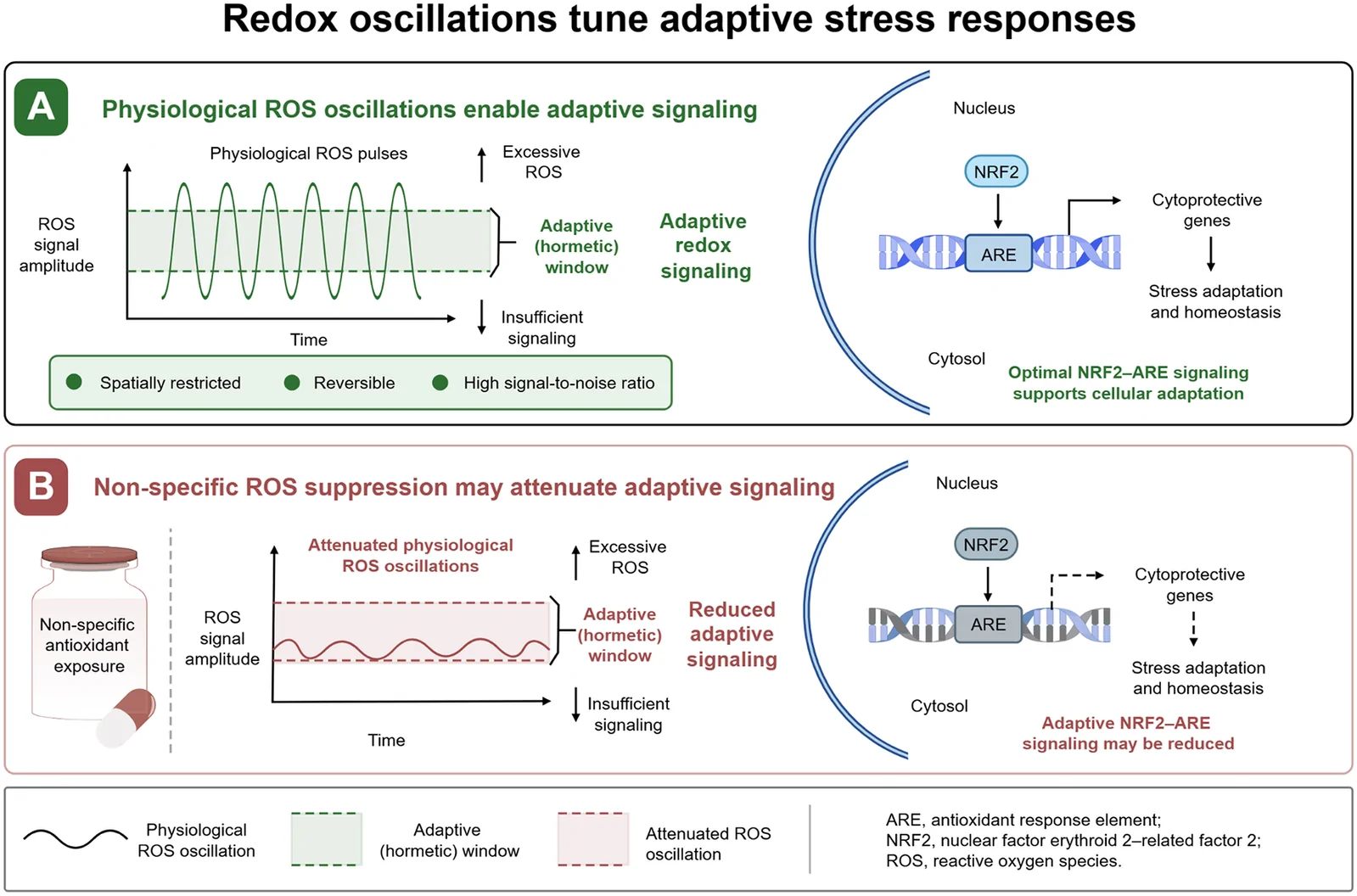
Temporally organized redox signaling and the risk of poorly targeted antioxidant suppression. Physiological redox information may be encoded as stimulus-evoked pulses, recurrent oscillations, circadian rhythms, or appropriate recovery kinetics. The oscillatory trace shown here is an illustrative conceptual pattern rather than a claim that all immune ROS signals are oscillatory. Experimental studies support endogenous H_2_O_2_ and peroxiredoxin redox rhythms and demonstrate reciprocal redox-clock coupling; recent aging work further links disruption and restoration of redox rhythms to functional phenotypes (55–59). Poorly timed or excessive antioxidant suppression may therefore attenuate adaptive redox dynamics in some contexts even when mean oxidant burden is lowered. This is an original conceptual schematic; no experimental data are shown. Created using Figdraw.

This argument does not imply that antioxidant strategies are intrinsically beneficial or harmful. Rather, the fidelity framework predicts that the outcome depends on whether an intervention selectively reduces pathological redox noise while preserving stimulus-coupled, appropriately routed, reversible, and adaptively decoded redox signaling. Antioxidants may be useful when oxidative stress is acute, when a pathological reactive-species source is well defined, or when treatment is delivered to the relevant compartment (85, 112). Senolytic or senomorphic strategies likewise need biomarker-guided deployment because reducing SASP burden and preserving immune surveillance may diverge (21, 115). Mitochondria-targeted antioxidants, context-specific NOX modulation, NRF2 pathway tuning, support for glutathione/thioredoxin/peroxiredoxin systems, and mitophagy restoration should therefore be evaluated according to whether they improve redox organization, reversibility, and adaptive immune responsiveness, not only whether they reduce oxidant abundance.

Future trials should not stratify interventions solely by oxidative-stress burden. They should consider whether the dominant defect lies in source uncoupling or chemically inappropriate generation, encoding irreversibility, transmission misrouting or propagation failure, decoder mismatch, or resolution failure. This distinction may explain why similar redox-directed interventions yield different outcomes across age, tissue, disease stage, immune context, infection risk, vaccine state, and baseline adaptive reserve (**Box 2**).

Box 2 **When antioxidant interventions may still be beneficial.**

Antioxidant strategies are most plausible when oxidative stress is dominated by an identifiable source, when oxidant production is acutely excessive, or when intervention is targeted to the relevant compartment or pathway. Examples include mitochondria-targeted antioxidants, selective NOX modulation, and therapies that restore glutathione, thioredoxin, or peroxiredoxin buffering capacity. Benefit is more likely when treatment reduces pathological redox noise while preserving physiological ROS pulses, reversible encoding, and adaptive decoding.

## 10 Testing the model: decisive experiments and quantitative platforms

To move beyond conceptual synthesis, the fidelity model must be testable. The decisive experiment is not another bulk measurement of total ROS. It is a paired input-output experiment in young and aged cells in which a defined immune or metabolic stimulus is linked to a chemically appropriate redox readout, temporal dynamics, reversible molecular encoding, decoder activation, and functional output. A key discriminator from conventional oxidative stress is that fidelity can be impaired even when mean ROS abundance is unchanged; conversely, oxidative damage can increase while stimulus-specific coupling remains relatively preserved. The strongest design would also test whether an intervention restores organization without requiring global suppression of ROS.

A practical workflow would include: (1) young and aged cells or tissues; (2) a defined stimulus such as TCR engagement, BCR engagement, TLR activation, phagocytosis, mitochondrial substrate shift, or a controlled redox perturbation; (3) time-resolved and, where possible, species- or compartment-sensitive redox measurements; (4) predefined temporal features such as amplitude, baseline variance, phase/frequency when rhythmicity is relevant, and recovery kinetics; (5) cysteine redox proteomics or targeted biochemical assays to distinguish reversible from persistent modifications; (6) decoder readouts such as NRF2 nuclear translocation, AMPK/mTOR phosphorylation, NF-kappaB/NLRP3 activation, or interferon-linked output; (7) functional readouts such as immune activation, cytokine resolution, metabolic adaptation, pathogen clearance, antigen presentation, antibody production, or cell survival; and (8) orthogonal source or pathway perturbations with controls for pleiotropic metabolic effects.

Different platforms map onto different layers of the fidelity circuit. Genetically encoded sensors such as roGFP- and HyPer-based probes can report compartment-targeted redox dynamics, but they require careful calibration and do not directly provide a complete spatial trajectory of the initiating oxidant. Redox proteomics can resolve oxidized cysteine species and estimate selected oxidation states, while conventional bulk workflows remain coarse in time and subcellular origin; proximity-labeling approaches improve localization but retain technical limitations (63, 64, 82). Single-cell transcriptomics, proteomics, and metabolomics can profile decoder heterogeneity (23, 24, 95), and live-cell or super-resolution imaging can resolve selected microdomains and contact-site signals (83). Dynamical modeling may integrate these layers into prespecified coupling, SNR, routing, and reversibility metrics (116), but model parameters should remain tied to measurable quantities.

### 10.1 Practical design of human young-versus-aged studies

For human studies, a young-versus-aged comparison will usually be cross-sectional rather than a true same-donor comparison across decades. A practical design would recruit prespecified younger and older healthy cohorts and treat age as a between-donor factor, while pairing unstimulated and stimulated aliquots within each donor. Blood collection should be standardized for time of day, fasting status where relevant, anticoagulant, collection volume, venipuncture-to-processing interval, processing temperature, PBMC isolation, cryopreservation/thawing, operator, instrument settings, and stimulation conditions. Because PBMC composition changes with age, dynamic redox measurements should preferably be performed within a prespecified immune-cell population, or accompanied by cell-composition measurements, rather than assigning a bulk-PBMC difference directly to cell-intrinsic aging. The donor, not individual cells or wells, should remain the biological unit of inference. Repeat blood draws in a subset of participants can estimate short-term within-donor and harvesting variation. In animal studies, genetic background, sex, diet, housing, sampling time, and processing batch should likewise be matched, randomized, or modeled explicitly.

The expected variance is strongly endpoint dependent. In one PBMC study, DHE-based superoxide measurements showed intra- and inter-assay coefficients of variation (CVs) of approximately 5-7%; mitochondrial mass measured with MitoTracker Green showed intra-assay CVs of 3-10% and inter-assay CVs of 11-13%; and JC-1-based mitochondrial membrane-potential measurements showed intra-assay CVs of 6-12% and inter-assay CVs of 11-22% (117). On repeat sampling one week apart, superoxide and mitochondrial-mass measurements in PBMCs/lymphocytes showed CVs of about 4-8%, whereas membrane-potential measurements were more variable (24-39% in PBMCs/lymphocytes and up to 57% in monocytes in that study) (117). Biological heterogeneity can be larger: analysis of cryopreserved PBMCs from 176 individuals found inter-individual CVs of 28.7-157% for different immune-cell population frequencies, with age and ethnicity contributing to variation in selected subsets (118). These values are assay- and population-specific, but they illustrate why variance should be estimated for each prespecified donor-level endpoint rather than assumed for ‘ROS’ as a single measurement.

Pre-analytical handling should therefore be treated as part of the experimental model. A classic processing study reported that PBMC isolation/cryopreservation at 24 h rather than within 8 h after venipuncture was associated with about an 8% reduction in viability, a 32% reduction in cell recovery, and a 36-56% reduction in antigen-specific IFN-gamma responses (119); a recent methodological review likewise emphasizes standardized and documented procedures for collection, processing, freezing, thawing, and stimulation (120). For planning, sample size should be based on pilot estimates of the donor-level effect and variance of the specific endpoint. As a simple orientation, a two-sided equal-sized two-group comparison at alpha=0.05 and 80% power requires approximately 26 evaluable donors per group for a standardized effect size of 0.8 and approximately 45 per group for an effect size of 0.6. These numbers are not prescriptive; paired within-donor stimulus-response measurements can improve precision, but they do not remove genuine between-person heterogeneity. Reporting both technical CVs and short-term within-donor variability would make the feasibility and interpretation of young-versus-aged redox-fidelity experiments much clearer.

### 10.2 Causal redox switches and genetic uncoupling

The strongest causal test is to uncouple redox sensing from the basal function of a candidate sentinel protein. An ideal redox switch should show stimulus-dependent and reversible modification at a defined residue, age- or state-dependent changes in its amplitude or kinetics, and a functional consequence that can be phenocopied or rescued by site-specific perturbation. Where feasible, a redox-insensitive mutant should preserve the protein’s core structural or catalytic role while selectively reducing responsiveness to the relevant redox/electrophilic input. KEAP1 provides a partial precedent: Cys151 substitution can preserve basal KEAP1-CUL3 ubiquitin-ligase activity while abolishing responses to selected electrophilic inducers, although KEAP1 contains multiple chemically distinct sensor cysteines and therefore illustrates the complexity of such experiments (45, 65, 121). More directly in aging, perturbation of redox-sensitive CLOCK Cys195 was recently shown to alter redox-rhythm-dependent aging phenotypes (59). Few proteins currently satisfy all of these criteria, so site-specific mutants should be treated as high-value causal tests rather than assumed proof of a simple one-residue mechanism.

These measurements yield falsifiable predictions. Aged immune cells should exhibit one or more prespecified defects, such as higher baseline variability, weaker stimulus-linked SNR, abnormal pulse or rhythmic structure, poorer reversibility, excessive spatial spread or delayed propagation, and less coherent decoder responses. Interventions that restore mitochondrial quality control, lysosomal function, membrane/contact-site integrity, metabolic redox coupling, or endogenous buffering should improve the relevant fidelity dimension and immune function even if total ROS changes only modestly. Conversely, if aged cells retain normal input-output coupling, temporal organization, reversible encoding, and decoder coherence despite oxidative damage, redox-fidelity loss should not be invoked as the central mechanism in that context.

## 11 Therapeutic implications: from antioxidant burden reduction to redox network restoration

A fidelity model reorients therapeutic priorities. The aim is not to eliminate ROS but to restore the capacity of immune cells and tissues to generate, interpret, and resolve redox signals appropriately. This aligns redox biology with precision immune geroscience: treatment choice should depend on the dominant fidelity defect rather than on a generic oxidative-stress label. Candidate strategies, fidelity targets, precision risks, translational value, and biomarker-based stratification criteria are mapped in **Table 2**.

**Table 2:** Therapeutic strategies mapped to redox information flow, translational criteria, and candidate stratification biomarkers.

Redox level	Candidate strategy	Fidelity target	Precision risk	Potential biomarkers/stratification criteria
Generation/source fidelity	ETC optimization, mitochondrial quality control, mitochondria-targeted redox modulation, context-specific NOX modulation.	Reduce unstructured leakage while preserving stimulus-coupled ROS production.	Wrong source or excessive suppression may impair antimicrobial defense or physiological signaling.	Mitochondrial ROS dynamics, NAD+/NADH and NADP+/NADPH state, OCR/ECAR, membrane potential, NOX activity, source-specific sensors.
Encoding/reversible chemistry	Support glutathione, thioredoxin, and peroxiredoxin systems; reinforce thiol relay capacity.	Maintain reversible cysteine chemistry and relay capacity.	Excessive buffering may flatten adaptive redox pulses.	GSH/GSSG, Trx/Prx oxidation state, NADP+/NADPH, reversible cysteine oxidation, protein carbonylation.
Transmission/spatial routing	Membrane stabilization, MAM/contact-site preservation, phagosome/lysosome support, compartment-targeted delivery.	Preserve appropriate target engagement, spatial selectivity, and propagation kinetics.	Specific *in vivo* targeting remains difficult and may differ by immune cell type.	Organelle-targeted redox sensors, target/off-target signal ratio, propagation delay, MAM integrity, lipid peroxidation, lysosomal pH, membrane stability.
Decoding/adaptive responsiveness	NRF2 tuning, AMPK/mTOR rebalancing, selective NF-kappaB/NLRP3 or cGAS-STING restraint.	Restore proportional pathway responses to defined redox inputs.	Chronic activation or suppression may cause pathway fatigue, immune suppression, or oncogenic risk.	NRF2 nuclear translocation, AMPK/mTOR phosphorylation, NF-kappaB/NLRP3 activation, IFN-linked output.
Resolution/quality control	Enhance mitophagy, autophagy, lysosomal function, efferocytosis, and proteostasis restoration.	Remove persistent ROS-generating substrates and oxidized cargo.	Overactivation may compromise cell survival or acute immune activation.	Mitophagy flux, autophagic flux, lysosomal activity, oxidized-cargo clearance, return-to-baseline kinetics.
Network/system recoupling	Exercise, senolytics, microbiome/metabolic interventions, patient stratification, vaccination-timing optimization.	Recouple cellular, tissue, and systemic inflammatory tone.	Pleiotropic effects complicate attribution of redox-specific mechanisms.	Single-cell redox heterogeneity, SASP/inflammatory cytokines, immune functional reserve, frailty or resilience phenotypes.

ETC, electron transport chain; GSH/GSSG, reduced/oxidized glutathione; IFN, interferon; MAM, mitochondria-associated membrane; NET, neutrophil extracellular trap; NOX, NADPH oxidase; OCR/ECAR, oxygen consumption rate/extracellular acidification rate; Prx, peroxiredoxin; ROS, reactive oxygen species; SASP, senescence-associated secretory phenotype; Trx, thioredoxin.

At the source level, therapies should mitigate unstructured mitochondrial or NOX-derived oxidant production while preserving stimulus-linked signaling. At the encoding level, interventions should maintain thiol relay systems and reversible cysteine chemistry while recognizing secondary electrophilic/metabolic mediators. At the transmission level, they should preserve membrane integrity, phagosome/lysosome function, organelle contact-site organization, and appropriate propagation kinetics rather than enforcing maximal confinement. At the decoding level, they should restore proportional pathway responsiveness rather than chronically activate or suppress stress programs. At the resolution level, they should promote autophagy, mitophagy, lysosomal function, efferocytosis, and repair systems so that redox and inflammatory signals can terminate.

Lifestyle interventions such as exercise, caloric restriction, dietary modulation, and vaccination-timing strategies may be especially instructive because they often act through patterned stress rather than global suppression (32, 114). They may transiently increase ROS while improving mitochondrial quality control, inflammatory resilience, and metabolic flexibility. The same logic should guide pharmacological geroprotectors and immunomodulators: the key question is whether they restore redox network organization and immune resilience.

A clinically useful precision approach would stratify patients by dominant redox-fidelity defects. For example, a phenotype dominated by mitochondrial leakage may require mitochondrial quality-control restoration, whereas a phenotype dominated by inflammatory decoder bias may require selective NF-kappaB/NLRP3 restraint. Patients with low adaptive reserve may benefit more from carefully dosed hormetic interventions than from strong scavenging. This stratification remains aspirational, but it provides a roadmap for biomarker-driven redox geroscience.

Evidence status: the therapeutic framework is intentionally translational and hypothesis-generating. Its clinical value will depend on whether redox-fidelity biomarkers can predict which patients require source modulation, relay support, decoder tuning, or resolution enhancement.

## 12 Controversies, limitations, and boundary conditions

A balanced redox-fidelity framework must recognize several controversies. First, ROS are not uniformly harmful or beneficial. NOX-derived ROS and mitochondrial ROS can support antimicrobial defense, T-cell activation, and metabolic adaptation, but can also drive tissue injury, inflammasome activation, and chronic inflammatory tone when mislocalized or poorly resolved. Second, NRF2 activation is often cytoprotective and anti-inflammatory, but excessive or chronic activation could suppress necessary immune responses or support survival of damaged or malignant cells. Third, inhibition of NLRP3 or cGAS-STING may reduce sterile inflammation in aging-related contexts, but could also impair host defense, antiviral immunity, vaccine responses, or antitumor surveillance. Fourth, the failure of broad antioxidant supplementation does not mean that all redox-directed therapies are ineffective; it means that timing, source, compartment, dose, and immune context matter. Recent reviews of immunosenescence and inflammaging underscore that immune-targeted interventions should be stratified by cell state, inflammatory context, and host-defense requirements rather than applied as uniform anti-inflammatory suppression (26–28).

Several boundary conditions are necessary if the framework is to remain testable. Many of its strongest claims are currently supported by converging evidence rather than by fully integrated aging experiments. Redox behavior is tissue-, cell-type-, sex-, microbiome-, infection-, and disease-stage dependent; an intervention that restores fidelity in one immune compartment may be neutral or harmful in another. ROS measurement remains technically challenging. Conventional dyes can be nonspecific, poorly localized, influenced by probe loading, metal ions, pH, light exposure, and redox-cycling artifacts, and may not distinguish superoxide, hydrogen peroxide, lipid peroxidation, or secondary oxidants reliably. Genetically encoded sensors also require careful interpretation: HyPer-based probes can be pH-sensitive, roGFP-based systems report specific redox couples depending on sensor design, and targeted probes may perturb the microdomain they are intended to measure. No current platform reconstructs the complete intracellular path of a reactive species with simultaneous molecular, spatial, and temporal resolution. Redox proteomics provides complementary residue-level information, but bulk workflows are limited in temporal and compartmental resolution; even localized approaches require rapid quenching, efficient alkylation, preservation of native oxidation states, and careful distinction between reversible modifications and irreversible lesions (82).

These limitations define the field’s next experiments. A strong test of the model should specify what would falsify it. If aged immune cells show preserved compartment-specific redox SNR, normal reversibility, and coherent decoder responses despite oxidative damage, then redox-fidelity loss would not be a central mechanism in that context. If broad antioxidant suppression consistently restores immune function without preserving redox dynamics, the therapeutic premise would need revision. If immune decline proceeds independently of redox source coupling, reversible encoding, and resolution kinetics in a given model, then the fidelity framework should be restricted to other contexts rather than generalized.

## 13 Conclusions

Immunosenescence and inflammaging should not be understood exclusively as consequences of oxidative injury accumulation. In selected biological contexts, they may also reflect progressive loss of redox information quality. In young or resilient immune systems, redox signals are generated at defined sources, encoded by reversible or interpretable chemistry, transmitted with sufficient spatial and temporal selectivity, decoded by appropriate pathways, and resolved by buffering and quality-control mechanisms. With age, these layers may become noisy, mistimed, misrouted, insufficiently propagated, persistent, or poorly coupled. We propose that age-associated loss of redox signaling fidelity offers a testable framework for linking mitochondrial dysfunction, autophagy decline, altered antigen responses, myeloid inflammatory bias, cellular senescence, and chronic inflammatory propagation.

The translational implication is important but should be framed precisely: future therapies should aim to restore redox organization rather than indiscriminately remove ROS. The most informative biomarkers are likely to measure compartment-specific dynamics, SNR, reversibility, decoder proportionality, and resolution kinetics. Likewise, the most informative interventions will be those that restore source specificity, reversible buffering, spatial routing, inflammatory resolution, and immune network coupling. By linking biomarkers and interventions to the same fidelity dimensions, this hypothesis-generating framework may help shift aging immunology from oxidative-damage accounting toward precision immune geroscience.
